# Supplementary material for: Inhibition of type 1 immunity with tofacitinib is associated with marked improvement in longstanding sarcoidosis
Source: Nat Commun. 2022 Jun 6;13:3140. doi: 10.1038/s41467-022-30615-x (PMC9170782; doi:10.1038/s41467-022-30615-x)
Supplement: Supplementary file 1 — Supplementary Information [file 41467_2022_30615_MOESM1_ESM.pdf]

Inhibition of type 1 immunity with tofacitinib is associated with improvement in longstanding sarcoidosis

William Damsky[1,2], Brett King[2], and co-authors

[1]First author
[2]Corresponding authors

Supplementary Figures

a

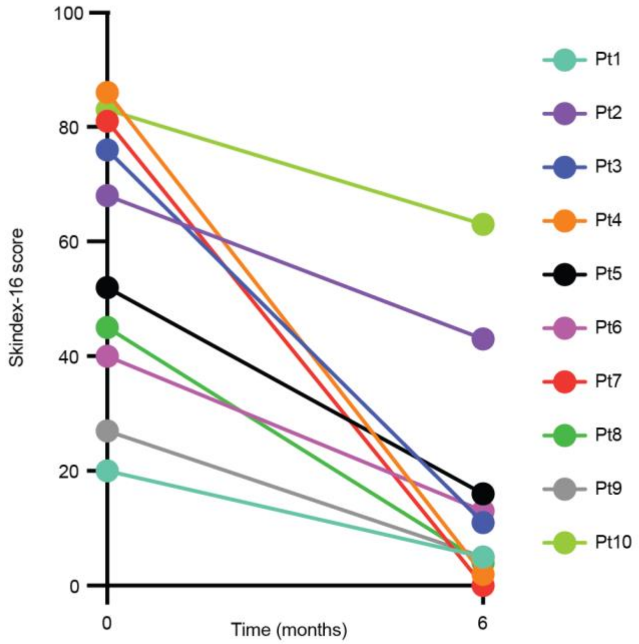

Supplementary Fig. 1. Changes in skin-related quality of life metric (Skindex-16).
a The Skindex-16 metric was administered at baseline and again after 6 months of tofacitinib. Source data are provided as a Data Source File.

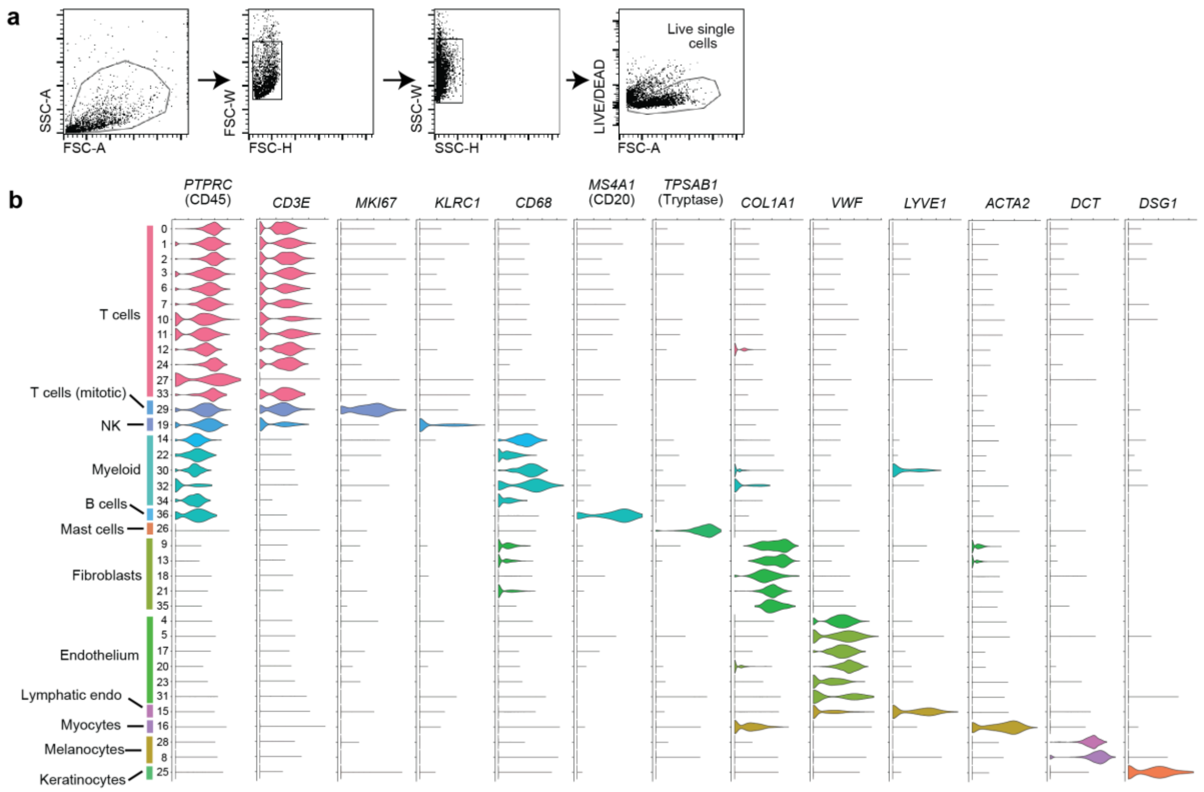

Supplementary Fig. 2. FACS gating strategy and lineage markers used for analysis of scRNA-seq data from sarcoidosis and control skin. a Gating strategy used for cell sorting to purify live single cells from dissociated skin samples. LIVE/DEAD Red viability dye was measured in the PE-Texas Red channel. **b** Violin plots showing cell-lineage markers used to classify cell types in cutaneous scRNA-seq data; corresponding to **Figure 3a-c.**

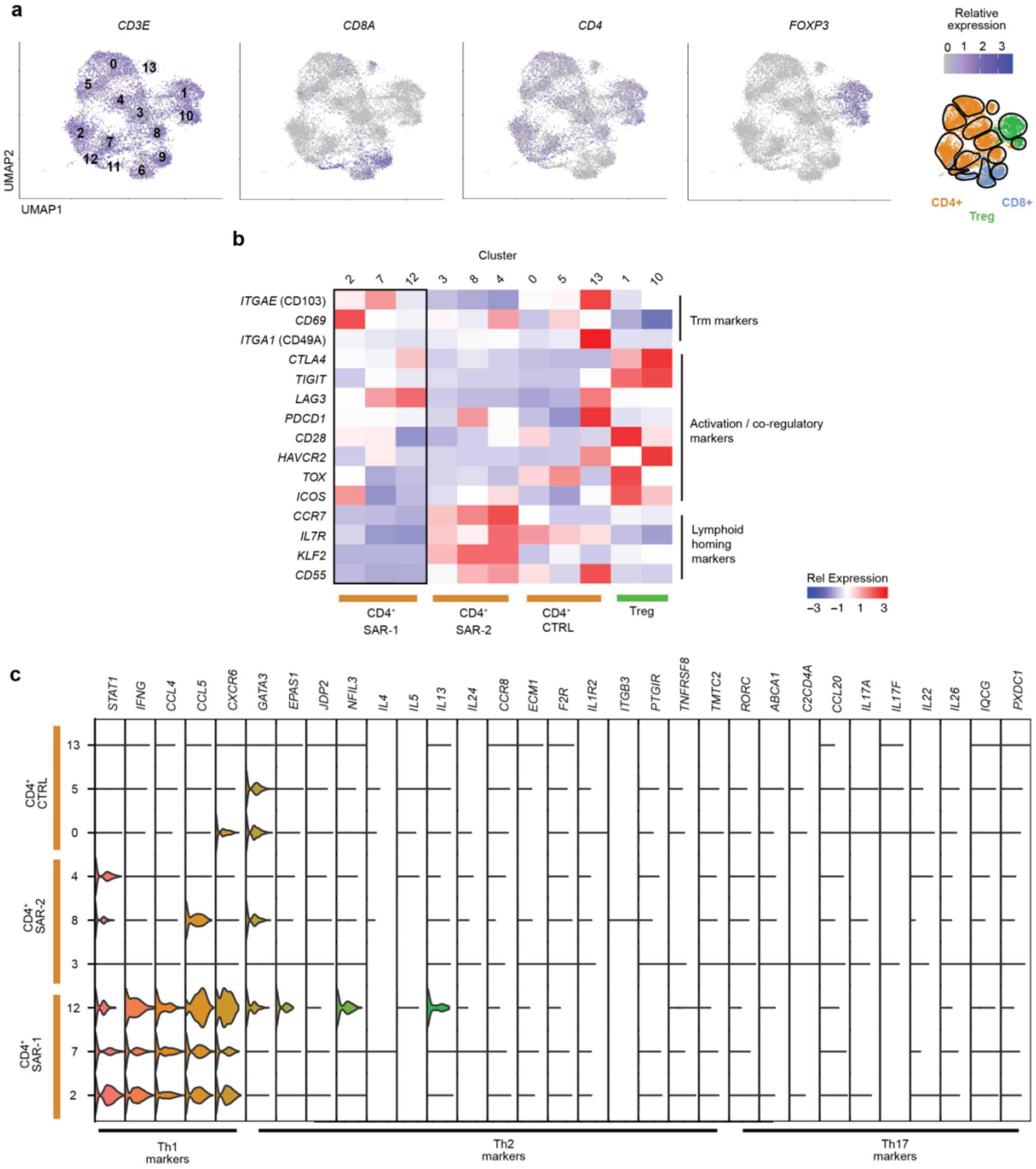

Supplementary Fig. 3. Analysis of T cells in scRNA-seq experiments from skin. a UMAP projections of T cell clusters from scRNA-seq experiments (corresponding to **Figure 4a**) showing relative expression of selected genes. **b** Heatmap showing expression of selected genes in CD4$^+$ T cell populations, including Tregs (*CD4*$^+$*FOXP3*$^+$). **c** Violin plots showing expression of select genes in CD4$^+$ T cell populations.

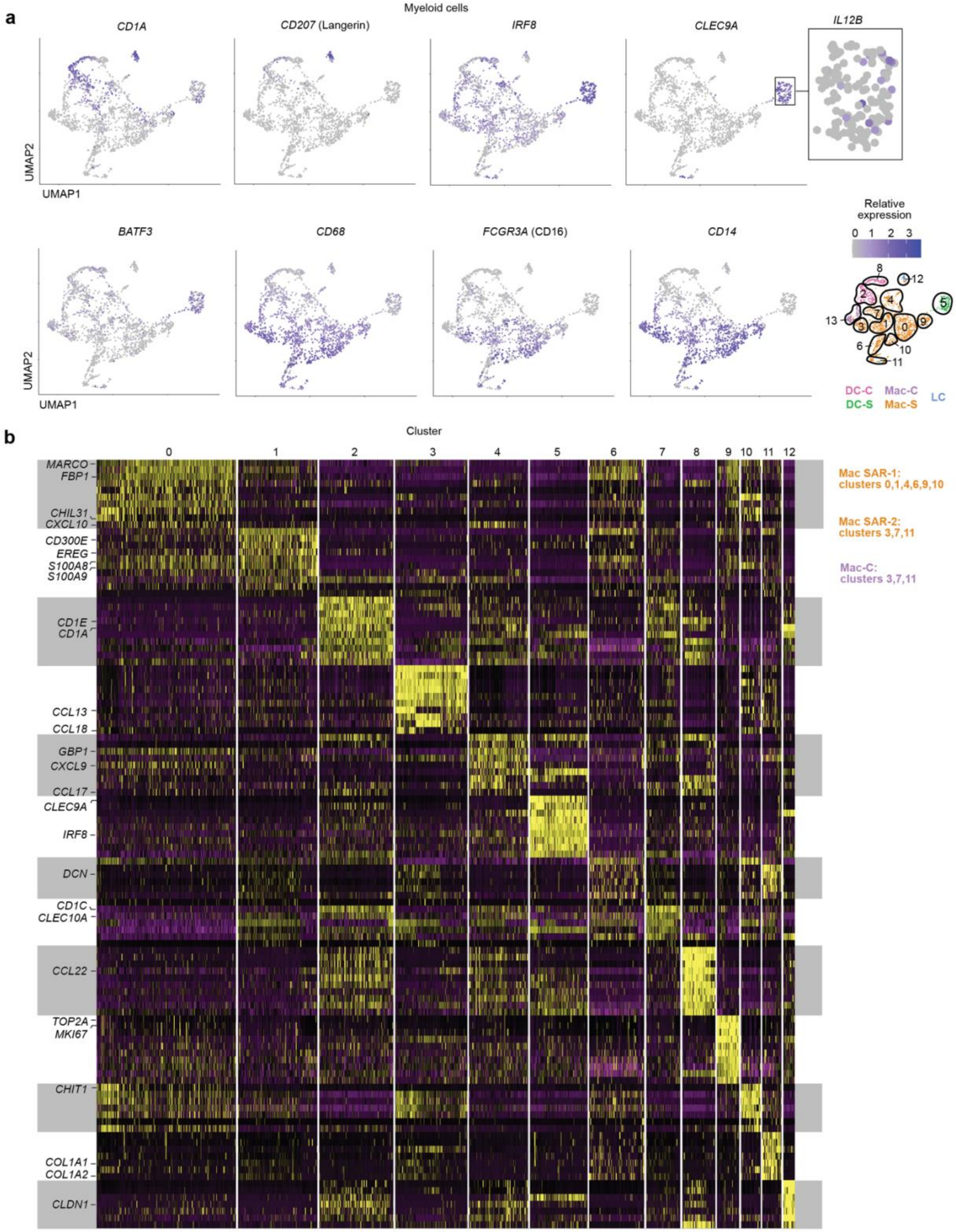
Myeloid cells
a
CD1A
CD207 (Langerin)
IRF8
CLEC9A
IL12B
UMAP2
UMAP1
BATF3
CD68
FCGR3A (CD16)
CD14
Relative expression
0 1 2 3
DC-C
Mac-C
DC-S
Mac-S
LC
b
Cluster
0 1 2 3 4 5 6 7 8 9 10 11 12
MARCO
FBP1
CHIL31
CXCL10
CD300E
EREG
S100A8
S100A9
CD1E
CD1A
CCL13
CCL18
GBP1
CXCL9
CCL17
CLEC9A
IRF8
DCN
CD1C
CLEC10A
CCL22
TOP2A
MKI67
CHIT1
COL1A1
COL1A2
CLDN1
Mac SAR-1: clusters 0,1,4,6,9,10
Mac SAR-2: clusters 3,7,11
Mac-C: clusters 3,7,11

Supplementary Fig. 4. Analysis of myeloid cells in scRNA-seq experiments from skin. a UMAP projections of myeloid cell clusters from scRNA-seq experiments (corresponding to **Figure 4f**) showing relative expression of selected genes used for cell type identification. Inset shows *IL12B* expression in cluster DC-S (DCs with a cDC1 phenotype in sarcoidosis). **b** Heatmap showing expression of differentially expressed genes among the myeloid clusters; yellow: upregulated, purple: downregulated.

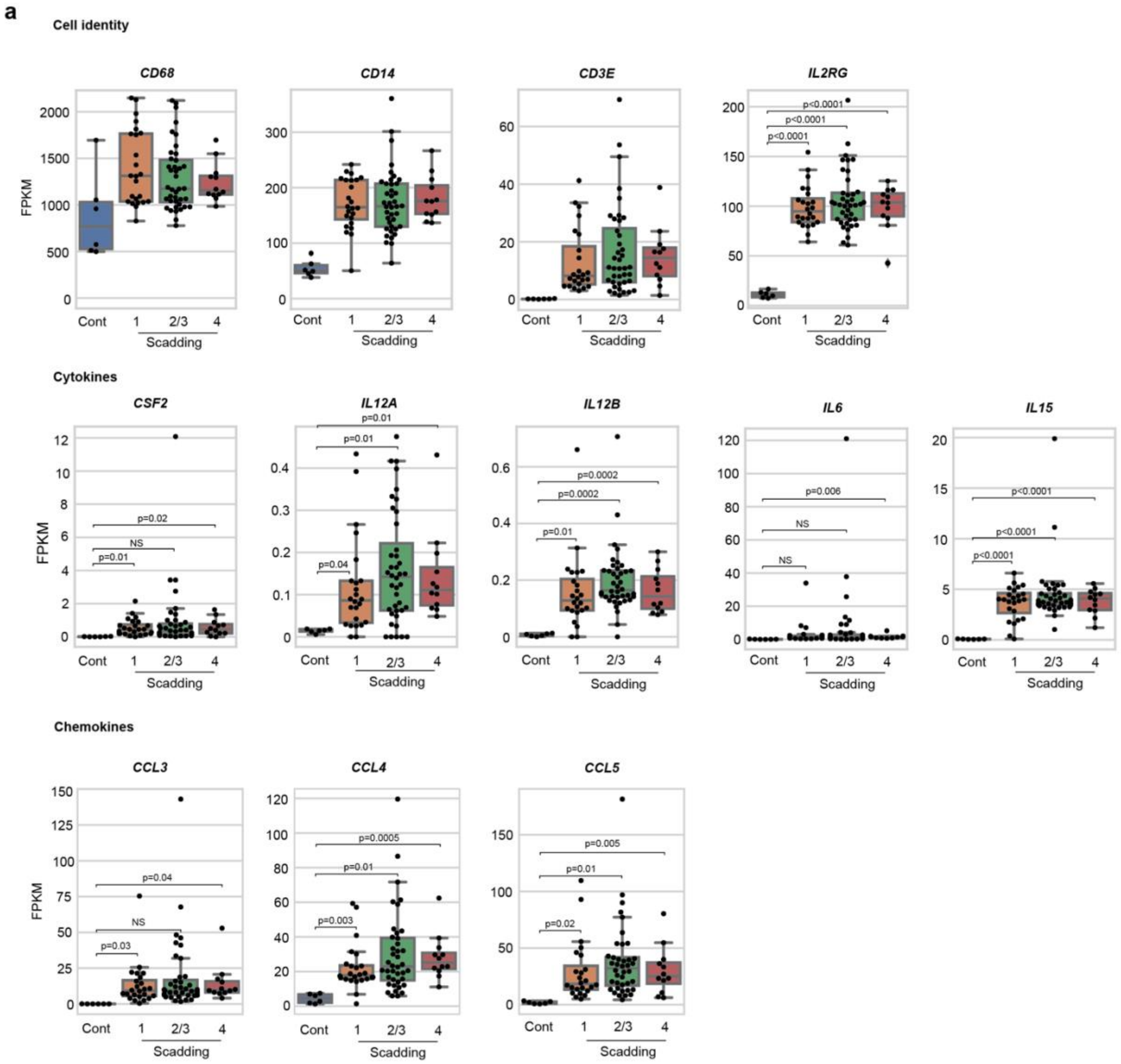

Supplementary Fig. 5. Analysis of bulk RNAseq data of bronchoalveolar lavage (BAL). Patients from the GRADS study[1] with sarcoidosis including Scadding stage 1 (n=24), stage 2/3 (n=40), and stage 4 (n=12) disease and 6 healthy control patients [2] were included. **a** Box and whisker plots showing gene expression for selected genes across samples according to Scadding stage and relative to controls. Box plots indicate median (middle line), 25th, 75th percentile (box), and 5th and 95th percentile (whiskers), p value determined using unpaired t-tests. Source data are provided as a Data Source File.

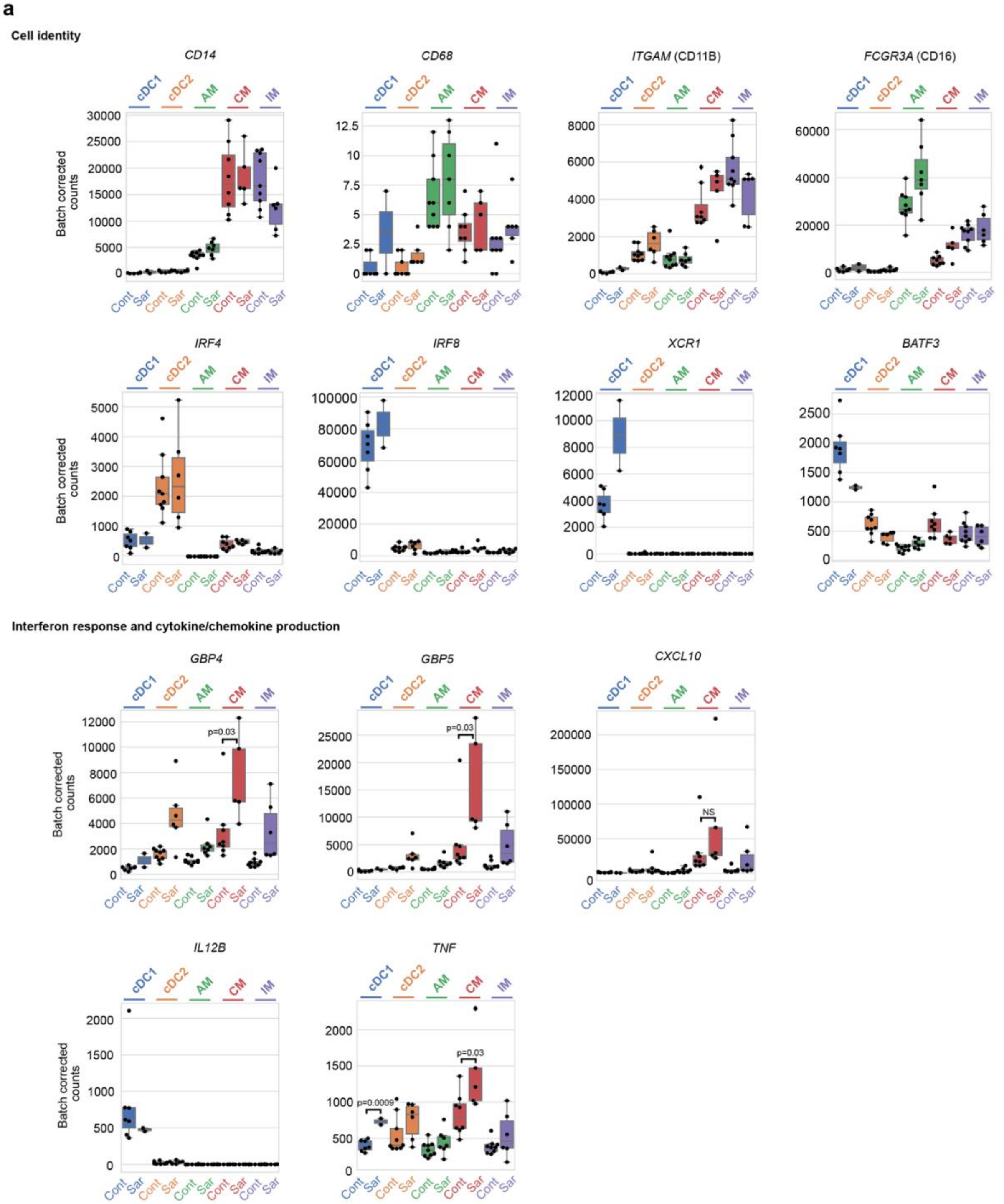
a
Cell identity
CD14
CD68
ITGAM (CD11B)
FCGR3A (CD16)
IRF4
IRF8
XCR1
BATF3
Interferon response and cytokine/chemokine production
GBP4
GBP5
CXCL10
IL12B
TNF
cDC1
cDC2
AM
CM
IM
Cont
Sar
Batch corrected counts
p=0.03
p=0.0009
NS

Supplementary Fig. 6. Analysis of RNAseq data from FACS purified myeloid populations from bronchoalveolar lavage (BAL). Samples from patients with sarcoidosis (n=8) and healthy controls (n=9) were included[3] (all available data was included). **a** Box and whisker plots showing gene expression patterns for selected genes; median (middle line), 25th, 75th percentile (box), and 5th and 95th percentile (whiskers), p value determined using unpaired t-tests. NS: not significant, AM: alveolar macrophages, CM: classical monocytes, IM: intermediate monocytes. Cells were stimulated with 1 µg/mL lipopolysaccharide (LPS) prior to analysis[3]. Source data are provided as a Data Source File.

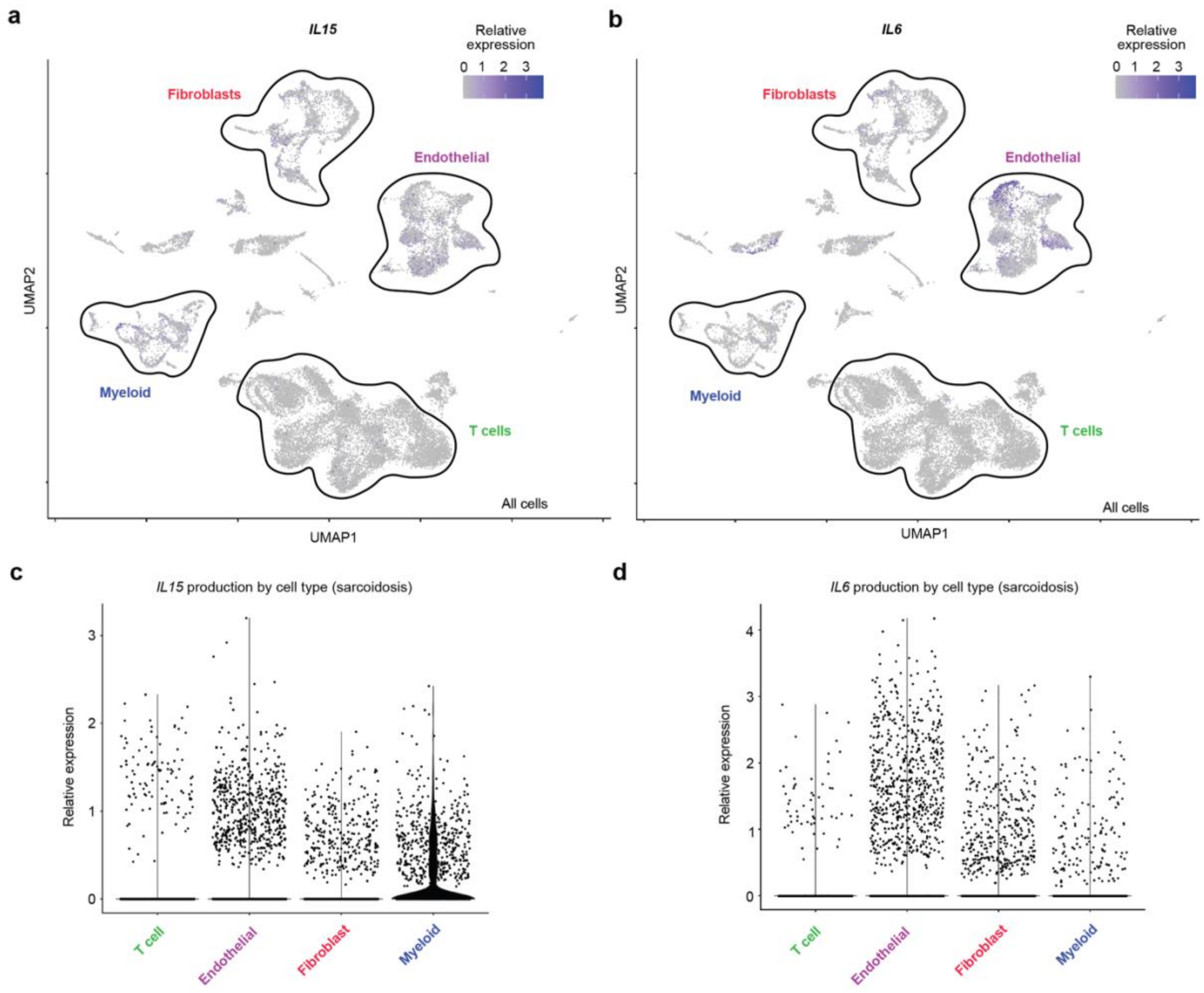

Supplementary Fig. 7. Analysis of all cells in skin scRNA-seq experiments. a UMAP projection of all cells from skin scRNA-seq experiments (corresponding to **Figures 3a-c and 8a**) showing relative expression of *IL15*. **b** UMAP projection of all cells from skin scRNA-seq experiments (corresponding to **Figures 3a-c and 8a**) showing relative expression of *IL6*. **c** Violin plot showing relative expression of *IL15* by cell type in sarcoidosis libraries. **d** Violin plot showing relative expression of *IL6* by cell type in sarcoidosis libraries.

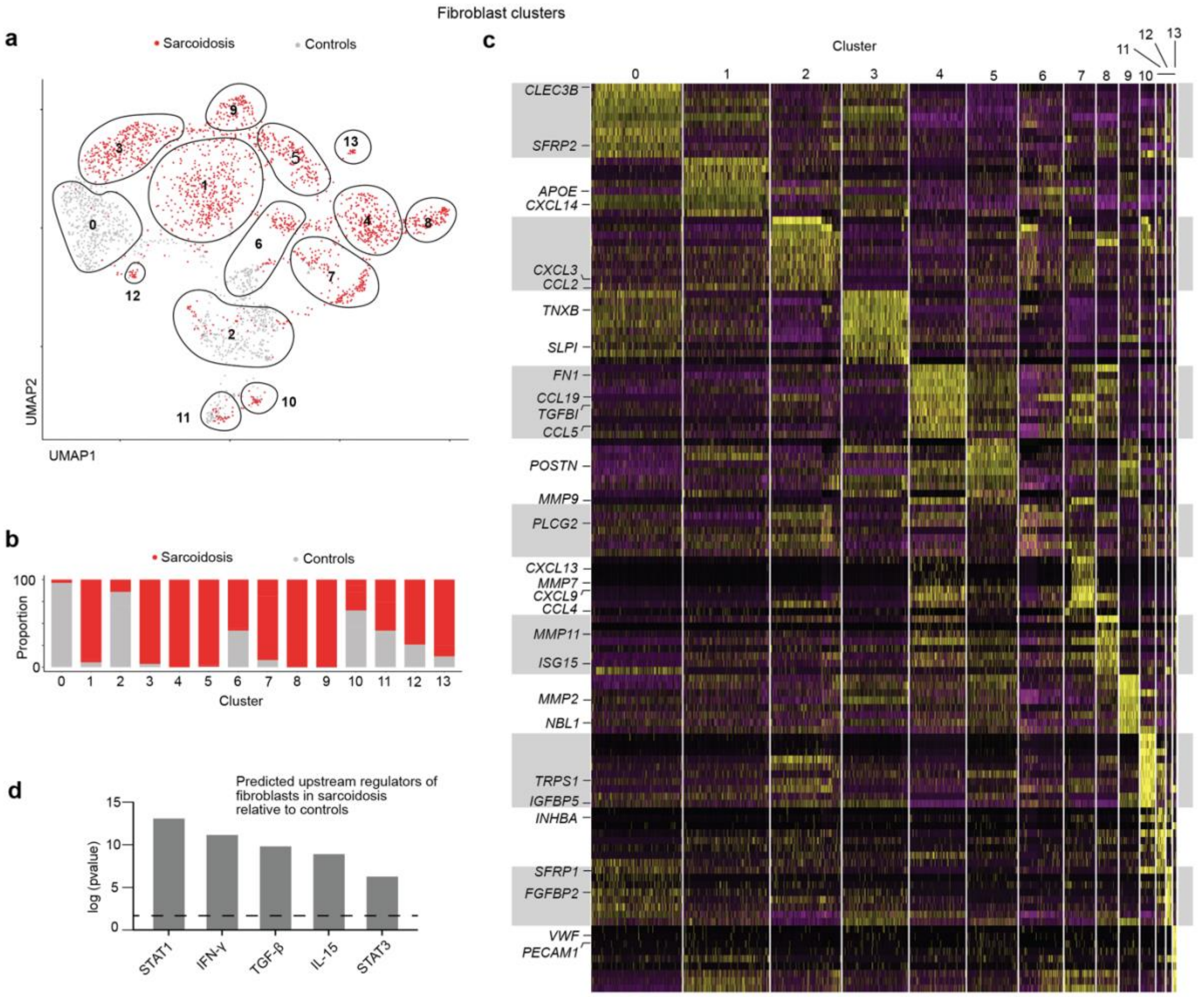

Supplementary Fig. 8. Analysis of fibroblast clusters in skin scRNA-seq experiments. a UMAP projection of scRNA-seq data showing fibroblast clusters in sarcoidosis compared to controls. **b** Histograms showing contribution of each condition (sarcoidosis: red, grey: controls) to each fibroblast cluster. **c** Heatmap showing differentially expressed genes in fibroblast clusters; yellow: upregulated, purple: downregulated. **d** Histogram showing selected predicted upstream regulators in fibroblasts in sarcoidosis (clusters 1, 3, 4, 5, 6, 7, 8, and 9) versus fibroblasts in controls (clusters 0 and 2) as determined by IPA. Significance cutoff of $p<0.001$ is shown by a dotted horizontal line and determined using Fisher exact test, right-tailed.

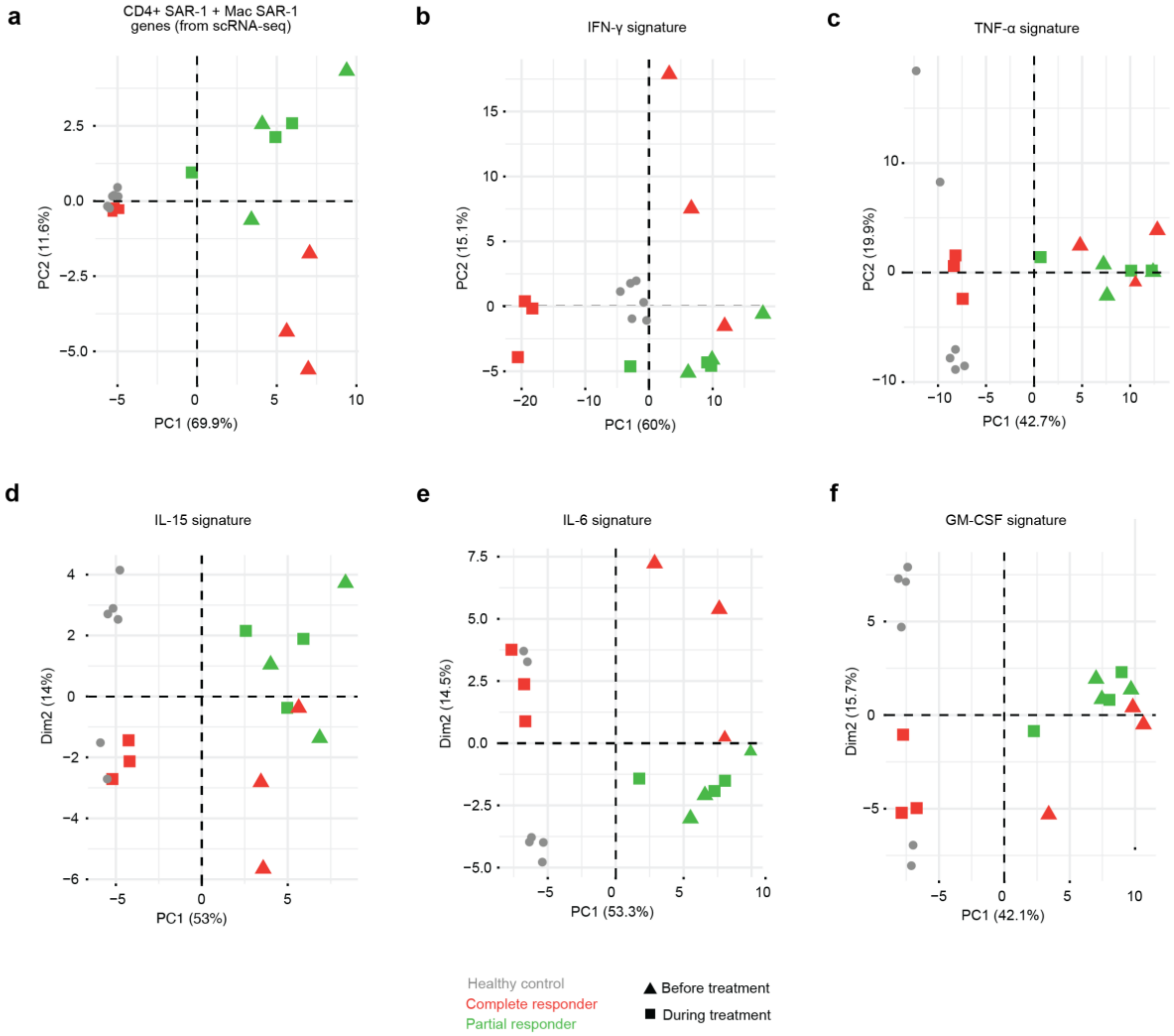

Supplementary Fig. 9. Principal component analysis of bulk skin RNA-seq data with various gene sets as a function of treatment. Analysis of gene expression data from skin in complete responders (CR) and partial responders (PR) relative to healthy controls. **a** Macrophage (Mac SAR-1) and T cell ($CD4^{+}$ SAR-1) activation signature genes are the same as those from scRNA-seq analysis (**Figure 4d, 4i**) and were used to perform principal component analysis. **b-f** Cytokine response signature gene sets (same as in **Figure 9a**) were also used to perform principal component analysis.

Supplementary Tables

Supplementary Table 1. Characteristics of pulmonary involvement in study patients. FEV1%: forced expiratory volume in 1 second (% of predicted), FVC%: forced vital capacity (% of predicted), DLCO-Hgb% Diffusion capacity for carbon monoxide adjusted for hemoglobin, N/A: data was unavailable.

Patient	Scadding Stage	Pre FEV1%	Pre FVC%	Pre FEV1/FVC	Pre DLCO-Hgb%
1	2	N/A	N/A	N/A	N/A
2	1	98	107	73	95
3	2	120	112	85	110
4	1	78	78	79	76
5	2	98	97	77	97
6	2	93	99	74	63
7	1	96	94	81	67
8	2	79	83	66	70
9	0	N/A	N/A	N/A	N/A
10	2	125	125	79	84

Supplementary Table 2. Summary of adverse events.
*This was a chronic, pre-existing condition. The Achilles tendon was not significantly PET avid, arguing against this being a specific manifestation of sarcoidosis. **The patient requested this approach as it had worked well for him in the past.

Adverse event	Incidence	Outcome	Serious AE?
SARS-CoV-2 infection	2	resolved, inpatient care not required	no
Acute sinusitis	1	treated with oral antibiotics	no
Lymphopenia	1	transient	no
Elevated LFTs	1	transient	no
Hyperlipidemia	1	statin initiated	no
Weight gain	1	persisted	no
Worsening Achilles tendinopathy pain*	1	treated with prednisone**	no

Supplementary Table 3. Samples used for scRNA-seq experiments.
Treatments were stable and sarcoidosis was clinically active (e.g. granulomas were still present in the skin) at the time of biopsy despite treatment.

**Healthy controls**				
Sample	Age	Sex	Location	Treatment
1	69	F	arm	None
2	37	F	arm	None
3	40	M	arm	none
**Sarcoidosis patients**				
Sample	Age	Sex	Location	Treatment
Pt1	63	F	arm	None
Pt2	55	F	arm	Hydroxychloroquine
Pt5	57	M	arm	methotrexate + prednisone

Supplementary Table 4. Differentially expressed genes in CD8$^+$ T cells in the cutaneous sarcoidosis scRNA-seq data. Comparison of the most differentially expressed genes between CD8$^+$ T cells from sarcoidosis libraries and control libraries. Low number of CD8$^+$ T cells in normal skin limited interpretation of this comparison.

Upregulated in sarcoidosis CD8$^+$ T cells				Upregulated in control CD8$^+$ T cells		
Gene	Avg_log2FC	Adj p value		Gene	Avg_log2FC	Adj p value
RPS26	2.25	2.43E-32		*IRTM1*	-1.01	6.51E-09
XIST	1.78	1.74E-10		*SQSTM1*	-1.02	9.13E-05
COTL1	0.94	0.00831		*FOSL2*	-1.03	9.01E-05
JUNB	0.81	0.0273		*ATP2B1*	-1.04	0.000316
RPS4X	0.59	4.75E-07		*ACO58791.1*	-1.07	0.00517
RPLP1	0.53	8.82E-07		*LMNA*	-1.18	0.00460
RPS19	0.53	8.29E-05		*ANKRD28*	-1.18	0.000502
RPL9	0.43	0.0479		*DOX3Y*	-1.28	2.59E-67
B2M	0.41	4.79E-08		*RPS4Y1*	-1.30	6.40E-48
RPL28	0.38	0.0283		*MTRNR2L1*	-1.38	2.09E-65
				CRIP1	-1.51	5.23E-11
				MTRNR2L12	-1.62	2.49E-16
				XCL2	-1.67	0.0410
				GNLY	-1.98	3.58E-13

Supplementary Table 5. Samples used for skin bulk RNA-seq experiments. HCQ: hydroxychloroquine, pred: prednisone, MTX: methotrexate, MMF: mycophenolate. CSAMI: cutaneous sarcoidosis activity and morphology instrument activity score. Pre-Tx: baseline treatment regimen on which the biopsy was obtained. Post-Tx: obtained while taking tofacitinib 5 mg twice daily. For the previously published patients, archival frozen tissue was processed, sequenced, and analyzed *de novo* as part of this study.

**Healthy volunteers**					
**Label**	**Age**	**Sex**	**Location**	**Reference**	**GEO Accession**
1	37	F	arm		
2	40	M	arm		
3-6	previously published			PMID: 30528824	GSE122592

**Sarcoidosis patients**										
**Label**	**Sample ID**	**Age**	**Sex**	**Location**	**Dur (yr)**	**Pre CSAMI**	**Pre Tx**	**Post CSAMI**	**Response**	**Reference**
A	SAR-A	48	F	back	8	85	none	0	CR	PMID: 30586518
B	SAR-B	34	M	back	6	68	none	0	CR	PMID: 31185230
Pt7	SAR-C	56	M	back	12	45	none	0	CR	
Pt2	SAR-D	55	F	arm	6	45	HCQ	22	PR	
Pt5	SAR-E	57	M	arm	22	55	MTX + pred	33	PR	
Pt8	SAR-F	55	M	arm	6	21	pred	6	PR	

Supplementary Table 6. Archival biopsy tissue used for RNA in situ hybridization studies. Lung: signifies lung parenchyma.

Tissue type	Condition	Age	Sex	Location	Method
Lung	sarcoidosis	64	M	L upper lobe	transbronchial
Lung	sarcoidosis	37	F	L upper lobe	transbronchial
Lung	sarcoidosis	88	F	R middle lube	transbronchial
Lung	sarcoidosis	45	F	L upper lobe	transbronchial
Lung	sarcoidosis	57	F	R lower lobe	wedge biopsy
Lung	sarcoidosis	39	M	L upper lobe	wedge resection
Lung	sarcoidosis	42	F	L upper lobe	wedge
Lung	sarcoidosis	72	F	R lung	pneumonectomy
Lung	sarcoidosis	79	M	L lower lobe	wedge resection
Lung	sarcoidosis	42	M	R middle lobe	transbronchial
Lung	adjacent normal	57	F	R lower lobe	wedge biopsy
Lung	adjacent normal	39	M	L upper lobe	wedge resection
Lung	adjacent normal	42	F	L upper lobe	wedge
Lung	adjacent normal	72	F	R lung	pneumonectomy
Lung	adjacent normal	79	M	L lower lobe	wedge resection
Skin	sarcoidosis	45	M	scalp	skin biopsy
Skin	sarcoidosis	35	M	back	skin biopsy
Skin	sarcoidosis	60	M	upper arm	skin biopsy
Skin	sarcoidosis	46	F	arm	skin biopsy
Skin	sarcoidosis	70	F	back	skin biopsy
Skin	sarcoidosis	49	M	scalp	skin biopsy
Skin	sarcoidosis	70	F	back	skin biopsy
Skin	sarcoidosis	49	F	upper back	skin biopsy
Skin	sarcoidosis	30	M	forehead	skin biopsy
Skin	sarcoidosis	69	F	back	skin biopsy
Skin	Healthy controls (n=10)	Wang et al. 2021[4]			
Skin	Psoriasis controls (n=20)	Wang et al. 2021[4]			
Skin	Atopic dermatitis controls (n=26)	Wang et al. 2021[4]			

Supplementary Table 7. Characteristics of control samples used for plasma Olink protein analysis.

Healthy volunteers		
**Label**	**Age**	**Sex**
Healthy 1	73	F
Healthy 2	63	M
Healthy 3	57	F
Healthy 4	51	F
Healthy 5	52	F
Healthy 6	53	F
Healthy 7	55	F
Healthy 8	59	M
Healthy 9	58	F
Healthy 10	55	F
Healthy 11	60	F

Supplementary Table 8. Data available on Gene Expression Omnibus.

SuperSeries	GSE169149	
Bulk RNA-seq	GSE169146	Sample ID (corresponds to main text)
	GSM5176912	Sarcoidosis patient A prior to treated with tofacitinib
	GSM5176913	Sarcoidosis patient A after treated with tofacitinib for six months
	GSM5176914	Sarcoidosis patient B prior to treated with tofacitinib
	GSM5176915	Sarcoidosis patient B after treated with tofacitinib for six months
	GSM5176916	Sarcoidosis patient 7 prior to treated with tofacitinib
	GSM5176917	Sarcoidosis patient 7 after treated with tofacitinib for six months
	GSM5176918	Sarcoidosis patient 2 prior to treated with tofacitinib
	GSM5176919	Sarcoidosis patient 2 after treated with tofacitinib for six months
	GSM5176920	Sarcoidosis patient 5 prior to treated with tofacitinib
	GSM5176921	Sarcoidosis patient 5 after treated with tofacitinib for six months
	GSM5176922	Sarcoidosis patient 8 prior to treated with tofacitinib
	GSM5176923	Sarcoidosis patient 8 after treated with tofacitinib for six months
	GSM5176924	Healthy control 1
	GSM5176925	Healthy control 2
scRNA-seq	GSE169147	Sample ID (corresponds to main text)
	GSM5176926	Sarcoidosis patient 1
	GSM5176927	Sarcoidosis patient 2
	GSM5176928	Sarcoidosis patient 5
	GSM5176929	Healthy control 1
	GSM5176930	Healthy control 2
	GSM5176931	Healthy control 3
Proteomic	GSE169148	Sample ID (corresponds to main text)
	GSM5176932	Sarcoidosis patient 1 prior to treated with tofacitinib
	GSM5176933	Sarcoidosis patient 1 after treated with tofacitinib for six months
	GSM5176934	Sarcoidosis patient 2 prior to treated with tofacitinib
	GSM5176935	Sarcoidosis patient 2 after treated with tofacitinib for six months
	GSM5176936	Sarcoidosis patient 3 prior to treated with tofacitinib

	GSM5176937	Sarcoidosis patient 3 after treated with tofacitinib for six months
	GSM5176938	Sarcoidosis patient 4 prior to treated with tofacitinib
	GSM5176939	Sarcoidosis patient 4 after treated with tofacitinib for six months
	GSM5176940	Sarcoidosis patient 5 prior to treated with tofacitinib
	GSM5176941	Sarcoidosis patient 5 after treated with tofacitinib for six months
	GSM5176942	Sarcoidosis patient 6 prior to treated with tofacitinib
	GSM5176943	Sarcoidosis patient 6 after treated with tofacitinib for six months
	GSM5176944	Sarcoidosis patient 7 prior to treated with tofacitinib
	GSM5176945	Sarcoidosis patient 7 after treated with tofacitinib for six months
	GSM5176946	Sarcoidosis patient 8 prior to treated with tofacitinib
	GSM5176947	Sarcoidosis patient 8 after treated with tofacitinib for six months
	GSM5176948	Sarcoidosis patient 10 prior to treated with tofacitinib
	GSM5176949	Sarcoidosis patient 10 after treated with tofacitinib for six months
	GSM5176950	Sarcoidosis patient 1 treated with 5 mg tofacitinib
	GSM5176951	Sarcoidosis patient 3 treated with 15 mg tofacitinib
	GSM5176952	Healthy control 1
	GSM5176953	Healthy control 2
	GSM5176954	Healthy control 3
	GSM5176955	Healthy control 4
	GSM5176956	Healthy control 5
	GSM5176957	Healthy control 6
	GSM5176958	Healthy control 7
	GSM5176959	Healthy control 8
	GSM5176960	Healthy control 9
	GSM5176961	Healthy control 10
	GSM5176962	Healthy control 11

Supplementary References

1 Vukmirovic, M. *et al.* Transcriptomics of bronchoalveolar lavage cells identifies new molecular endotypes of sarcoidosis. *Eur Respir J* **58**, doi:10.1183/13993003.02950-2020 (2021).

2 Camiolo, M. J. *et al.* High-dimensional profiling clusters asthma severity by lymphoid and non-lymphoid status. *Cell Rep* **35**, 108974, doi:10.1016/j.celrep.2021.108974 (2021).

3 Lepzien, R. *et al.* Monocytes in sarcoidosis are potent tumour necrosis factor producers and predict disease outcome. *Eur Respir J* **58**, doi:10.1183/13993003.03468-2020 (2021).

4 Wang, A. *et al.* Cytokine RNA In Situ Hybridization Permits Individualized Molecular Phenotyping in Biopsies of Psoriasis and Atopic Dermatitis. *JID Innov* **1**, 100021, doi:10.1016/j.xjidi.2021.100021 (2021).
