# Supplementary material for: Inhibition of type 1 immunity with tofacitinib is associated with marked improvement in longstanding sarcoidosis
Source: Nat Commun. 2022 Jun 6;13:3140. doi: 10.1038/s41467-022-30615-x (PMC9170782; doi:10.1038/s41467-022-30615-x)
Supplement: Supplementary file 3 — Reporting Summary [file 41467_2022_30615_MOESM3_ESM.pdf]

nature research

Corresponding author(s): William Damsky

Last updated by author(s): 05/06/2021

Reporting Summary

Nature Research wishes to improve the reproducibility of the work that we publish. This form provides structure for consistency and transparency in reporting. For further information on Nature Research policies, see our Editorial Policies and the Editorial Policy Checklist.

Statistics

For all statistical analyses, confirm that the following items are present in the figure legend, table legend, main text, or Methods section.

n/a	Confirmed	
☐	☒	The exact sample size (*n*) for each experimental group/condition, given as a discrete number and unit of measurement
☒	☐	A statement on whether measurements were taken from distinct samples or whether the same sample was measured repeatedly
☐	☒	The statistical test(s) used AND whether they are one- or two-sided<br>*Only common tests should be described solely by name; describe more complex techniques in the Methods section.*
☒	☐	A description of all covariates tested
☒	☐	A description of any assumptions or corrections, such as tests of normality and adjustment for multiple comparisons
☐	☒	A full description of the statistical parameters including central tendency (e.g. means) or other basic estimates (e.g. regression coefficient) AND variation (e.g. standard deviation) or associated estimates of uncertainty (e.g. confidence intervals)
☐	☒	For null hypothesis testing, the test statistic (e.g. *F*, *t*, *r*) with confidence intervals, effect sizes, degrees of freedom and *P* value noted<br>*Give P values as exact values whenever suitable.*
☒	☐	For Bayesian analysis, information on the choice of priors and Markov chain Monte Carlo settings
☒	☐	For hierarchical and complex designs, identification of the appropriate level for tests and full reporting of outcomes
☒	☐	Estimates of effect sizes (e.g. Cohen's *d*, Pearson's *r*), indicating how they were calculated

Our web collection on statistics for biologists contains articles on many of the points above.

Software and code

Policy information about availability of computer code

Data collection	N/A
Data analysis	MIM version 7.0.4 software was used for analysis of PET-CT scans. MIM is a commercially available software. Partek Flow version 9.0 was used to process bulk RNAseq data. Partek Flow is a commercially available software. Burrows-Wheeler Aligner version 0.7.15 was used to align the bulk RNAseq reads. Burrows-Wheeler Aligner is a freely available software package. Cellranger software version 3.1.0 was used to process the single cell RNA sequencing data and is a commercially available software. Seurat version 3.2.0 is a freely available R package that was used to analyze the scRNA-seq data. Standard Seurat commands were used to perform the analysis and for data visualization. Additional freely available R scripts, ggplot (v3.3.3), ggrepl (v0.9.1), dplyr (v1.0.3), and heatmap.2 were utilized to create figures. Ingenuity Pathway Analysis content version 51963813 was used to perform pathway analysis, this is a commercially available software package. Cellphone DB version 2.1.4 is freely available and standard commands were used to perform the analysis. These approaches are described in detail in the supplementary methods section.

For manuscripts utilizing custom algorithms or software that are central to the research but not yet described in published literature, software must be made available to editors and reviewers. We strongly encourage code deposition in a community repository (e.g. GitHub). See the Nature Research guidelines for submitting code & software for further information.

Data

Policy information about availability of data

All manuscripts must include a data availability statement. This statement should provide the following information, where applicable:

- Accession codes, unique identifiers, or web links for publicly available datasets
- A list of figures that have associated raw data
- A description of any restrictions on data availability

scRNA-seq, bulk RNA-seq, and proteomic data have been uploaded to Gene Expression Omnibus (GSE169149). Details are provided in Supplementary Table 7.
Figures with associated raw data: Figure 3 and Supplementary Fig. 9, Supplementary Fig. 10, 2, S3, S4, S5,S6.
There are no restrictions on data availability.

Field-specific reporting

Please select the one below that is the best fit for your research. If you are not sure, read the appropriate sections before making your selection.

- [x] Life sciences
- [ ] Behavioural & social sciences
- [ ] Ecological, evolutionary & environmental sciences

For a reference copy of the document with all sections, see nature.com/documents/nr-reporting-summary-flat.pdf

Life sciences study design

All studies must disclose on these points even when the disclosure is negative.

Sample size	No sample size calculation was performed. This was designed to be a proof-of-concept study and enrolling 10 patients was arbitrary, but, we felt balanced ability to enroll the trial efficiently and also to assess activity of tofacitinib. For molecular analyses, sample sizes were determined by tissue availability.
Data exclusions	No data were excluded from the study. The clinical trial was performed under a blanket study which included 10 patients with sarcoidosis and 5 patients with granuloma annulare treated with the same intervention (NCT03910543). The 5 patients with granuloma annulare were analyzed and published separately because this is a distinct disorder with different primary and secondary outcome meaures.
Replication	Due to the clinical nature of the study, the findings cannot be reproduced within this study.
Randomization	This was an open label study, there was no randomization and all patients received the intervention.
Blinding	Blinding was not possible in this study as it was open-label, that is, all patients received the same intervention.

Reporting for specific materials, systems and methods

We require information from authors about some types of materials, experimental systems and methods used in many studies. Here, indicate whether each material, system or method listed is relevant to your study. If you are not sure if a list item applies to your research, read the appropriate section before selecting a response.

Materials & experimental systems

n/a	Involved in the study	
[ ]	[x]	Antibodies
[x]	[ ]	Eukaryotic cell lines
[x]	[ ]	Palaeontology and archaeology
[x]	[ ]	Animals and other organisms
[ ]	[x]	Human research participants
[ ]	[x]	Clinical data
[x]	[ ]	Dual use research of concern

Methods

n/a	Involved in the study	
[x]	[ ]	ChIP-seq
[x]	[ ]	Flow cytometry
[x]	[ ]	MRI-based neuroimaging

Antibodies

Antibodies used	CD68 (clone PG-M1, Agilent-Dako catalog # GA61361-2, lot # 20072835)
Validation	The staining was performed by a CLIA-certified clinical pathology laboratory.

Human research participants

Policy information about studies involving human research participants

Population characteristics	This information is summarized in Table 1. Patients all had a biopsy confirmed diagnosis of sarcoidosis with skin involvement. Patients ranged in age from 53-63, there were 6 males and 4 females, 6 patients had black or brown skin. Prior treatments are summarized in Table 1.
Recruitment	Participants were recruited from Yale Dermatology, Pulmonology, and Cardiology clinics. Patients with long-standing disease not controlled by standard therapies tended to be most commonly interested in participation.
Ethics oversight	This study was approved by the Yale University IRB.

Note that full information on the approval of the study protocol must also be provided in the manuscript.

Clinical data

Policy information about clinical studies

All manuscripts should comply with the ICMJEguidelines for publication of clinical research and a completedCONSORT checklist must be included with all submissions.

Clinical trial registration	NT03910543
Study protocol	The study protocol was submitted with and will be published alongside the manuscript as supplementary material.
Data collection	The data was collected at the Yale Center for Clinical Investigation at Yale University in New Haven, Connecticut, United States. Recruitment and data collection occurred from 4/2019 - 11/2020.
Outcomes	The predefined primary outcome was the change in Cutaneous Sarcoidosis Activity and Morphology Instrument (CSAMI) activity score after 6 months of treatment. CSAMI is a validated cutaneous sarcoidosis severity scoring tool. CSAMI utilizes a scoring rubric which was scored in person at the time of each visit. Secondary outcomes included: 1) change in Skindex-16: a skin-related quality of life metric after 6 months, 2) change in histologic findings in lesional skin after 6 months, 3) change in gene expression in lesional skin after 6 months, 4) change in plasma biomarkers after 6 months, and 5) change in activity of internal organ sarcoidosis (assessed with whole-body PET-CT) after 6 months. Skindex-16 is a validated skin-related quality of life metric which was administered at baseline and after 6 months. Histology and RNAseq changes were assessed by performing skin biopsies at baseline and after 6 months (patients with involvement of only cosmetically sensitive skin, e.g. the face, were not mandated to have these research biopsies performed). Plasma biomarkers were assessed with blood collection at baseline and after 6 months. Internal organ activity was assessed with whole body PET-CT scan at baseline and 6 months.
